# Suicide Prevention and Specialized Intervention: Structuring Actions in a Psychiatry and Chemical Dependency Service in Rio de Janeiro City

**DOI:** 10.1192/j.eurpsy.2023.2361

**Published:** 2023-07-19

**Authors:** J. A. Jaber Filho, A. D. Hollanda, V. Soares, P. Zanelatto, C. T. A. Garcia

**Affiliations:** 1 Saúde Mental, Clínica Jorge Jaber, Rio de Janeiro, Brazil

## Abstract

**Introduction:**

Suicide presents itself as a serious public health problem, with universal characteristics. Though world rates dropped between 2010 and 2016, they are still very high and in regions like the Americas there has been a raise in the same period in Brazil, contrary to the word trend and surpassing the avarage of the Americas (6%), the rate of suicides in each 100 thousand inhabitants has raised in about 7% in the period (WHO, 2019).

**Objectives:**

Considering the relevance of these aspects, this study presents the results of actions for treatment and prevention of suicidal behavior, developed by a psychiatry and chemical dependency inpatient service in the city of Rio de Janeiro.

**Methods:**

This showed the necessity of the creation of a specific program of suicidal treatment and prevention and the Institution established the following actions of intervention to hospitalized patients: permanent watching , reduction of access to instruments and methods to commit suicide, strengthening of the Life Appreciation Group (LAG) and the Cognitive Behavioral Therapy group, art therapy and physical activities.

**Results:**

It was observed that among the 370 patients hospitalized in the Institution in the period of the study, 137 presented suicidal behavior and only 2 died. From these two cases, one abandoned treatment and the other ocurred during the period of treatment.

**Conclusions:**

The developed program reached positive results in the intervention of the cases and the actions of prevention had expressive reach in the number of people, especially because the press promoted wide dissemination of information for immediate help.

**Disclosure of Interest:**

None Declared

